# Primary Cutaneous Cribriform Apocrine Carcinoma: A Case Report and Narrative Review

**DOI:** 10.3390/biomed5040026

**Published:** 2025-11-06

**Authors:** Robyn Okereke, Anthony Linfante

**Affiliations:** 1 Department of Dermatology, Oregon Health & Sciences University, Portland, OR 97239, USA; 2 Department of Dermatology, University of Texas Medical Branch at Galveston, Galveston, TX 77555, USA

**Keywords:** primary cutaneous cribriform apocrine carcinoma, primary, cutaneous, apocrine, cribriform

## Abstract

**Background and Clinical Significance:**

Primary Cutaneous Cribriform Apocrine Carcinoma (PCCAC) is a rare, inert low-grade cutaneous malignancy that is diagnosed on histopathologic assessment. PCCAC usually presents in middle-aged adults as a solitary, subcutaneous nodule on the extremities. Characterized by anastomosing tubules and solid/cribriform nests of atypical epithelial cells generating a sieve-like display, the tumor is a histopathological variant of apocrine metaplasia of the skin. PCCAC also follows characteristic staining patterns. It is important to distinguish PCCAC from other similar histological variants, which may hold more grievous indications.

**Case Presentation:**

A 47-year-old female presented with an enlarging, itchy growth of several months on her back. On physical exam, an indurated pink, nontender papule of 8 mm on the left lateral side wall was noted. **Histopathology** demonstrated a well-circumscribed, pandermal tumor composed of anastomosing solid and cribriform nests, tubules, and cords of mildly atypical, eosinophilic epithelial cells forming a glandular lumina. An immunohistochemical study revealed the tumoral epithelium to express CK7, CK5/6, BER-EP4, CD117 (C-kit), and S100. Positive EMA and CEA staining highlighted intratumoral glandular ductal differentiation and apocrine secretion. Immunohistochemical stains for CK20, GATA-3, and p63 were negative.

**Conclusions:**

We present this case to distinguish the histological attributes of PCCAC and help differentiate it from more concerning visceral metastatic malignancies. We follow with a narrative review of the histopathologic differential for PCCAC and feature reconciliation of corresponding staining patterns reported in the literature.

## 1. Introduction

Primary Cutaneous Cribriform Apocrine Carcinoma (PCCAC) is a rare, indolent, low-grade cutaneous malignancy that may represent a variant of apocrine carcinoma. First introduced in 1998, 46 cases of PCCAC were reported in English literature as of 2021, increasing to 59 cases published by 2022 [1,2]. PCCAC usually presents at the mean age of 47, as a non-tender, solitary, subcutaneous nodule on an extremity [1,2]. PCCAC has no known racial predilection [2]. The literature suggests that PCCAC may be more common in females [1,2]. PCCAC requires histopathologic assessment for definitive diagnosis. Histopathology will reveal anastomosing solid and cribriform nests, tubules, and cords of mildly atypical epithelial cells with characteristic staining patterns. Because alternative apocrine and eccrine gland derivatives may closely resemble PCCAC on histology, immunohistochemical (IHC) stains are important in distinguishing this entity.

## 2. Materials and Methods

All Immunohistochemical (IHC) staining was performed according to the manufacturers’ recommended protocols in the dermatopathology laboratory of University of Texas Medical Branch. Two board-certified dermatopathologists independently evaluated the immunohistochemically stained slides. A total of six tissue sections were examined, including both hematoxylin and eosin–stained and IHC-stained slides.

Positive staining was defined as cytoplasmic and/or membranous reactivity in *≥*10% of cells.

IHC staining was performed on formalin-fixed, paraffin-embedded tissue sections (4 µm thick). Sections were deparaffinized in xylene, rehydrated through graded alcohols, and subjected to heat-induced epitope retrieval using citrate buffer (pH 6.0) in a pressure chamber. Endogenous peroxidase activity was blocked with 3% hydrogen peroxide. Slides were incubated with the primary antibodies listed below, followed by visualization using a polymer-based detection system and diaminobenzidine (DAB) chromogen. Slides were counterstained with hematoxylin, dehydrated, and coverslipped.

Antibodies and Clones:

CK7 (clone OV-TL 12/30, Dako/Agilent, Santa Clara, CA, USA)

CK5/6 (clone D5/16B4, Dako/Agilent)

CK1/3 (clone AE1/AE3, Thermo Fisher Scientific, Waltham, MA, USA)

BerEP4 (clone Ber-EP4, Dako/Agilent)

EMA (clone E29, Dako/Agilent)

CEA (clone II-7, Leica Biosystems, Buffalo Grove, IL, USA)

CD117 (clone YR145, Cell Marque, Rocklin, CA, USA)

S100 (polyclonal, Dako/Agilent)

Mammaglobin (clone 304–1A5, Leica Biosystems)

D2–40 (clone D2–40, Dako/Agilent)

CAM5.2 (clone CAM5.2, BD Biosciences, San Jose, CA, USA)

p63 (clone 4A4, Dako/Agilent)

## 3. Case

A 47-year-old woman presented with an itchy, nontender, enlarging, indurated, pink 8 mm papule on the left lateral back of seven months duration. There were no findings concerning for malignancy nor consistent with dermatofibroma on dermoscopic examination.

**Figure 1.**
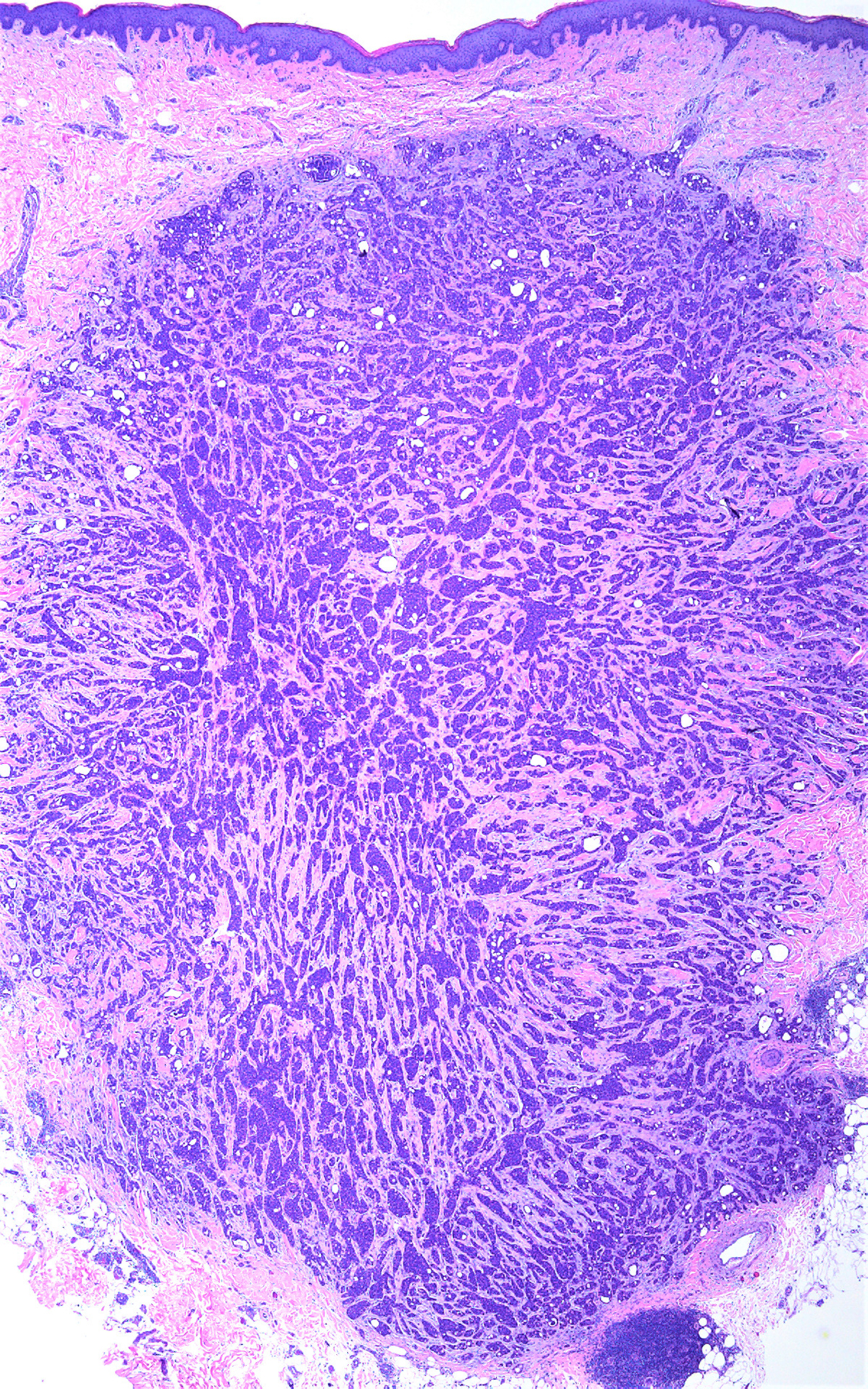

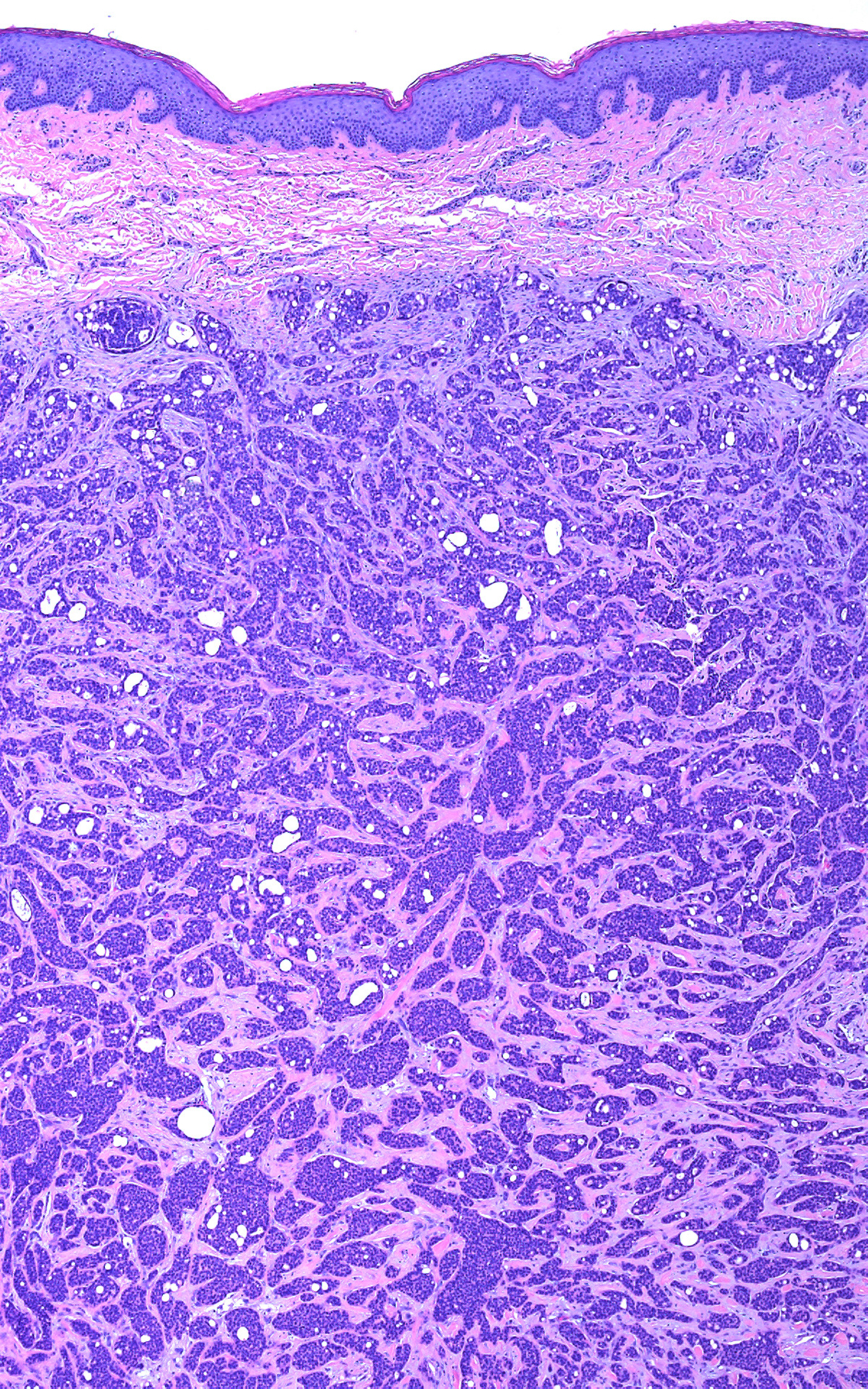

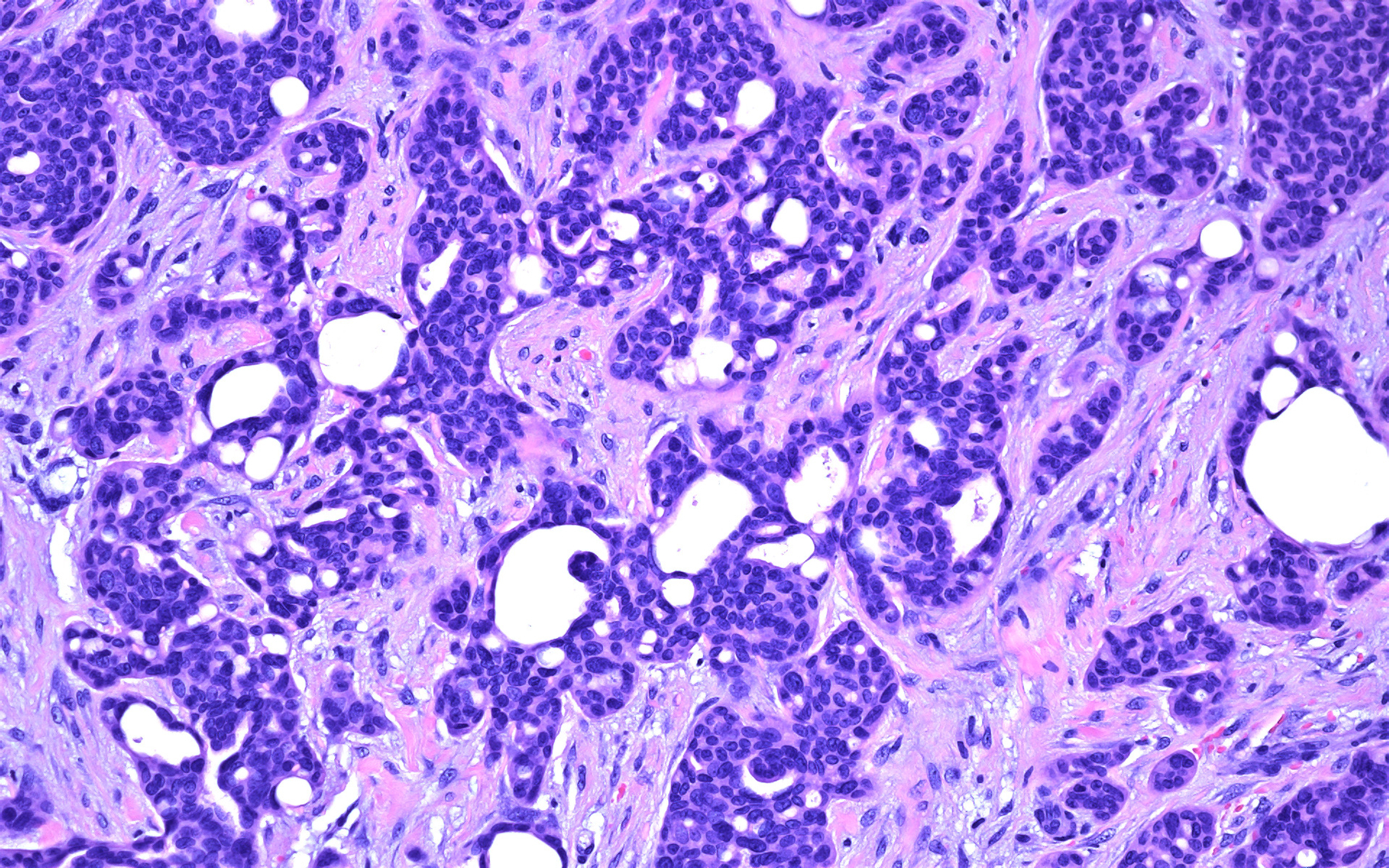

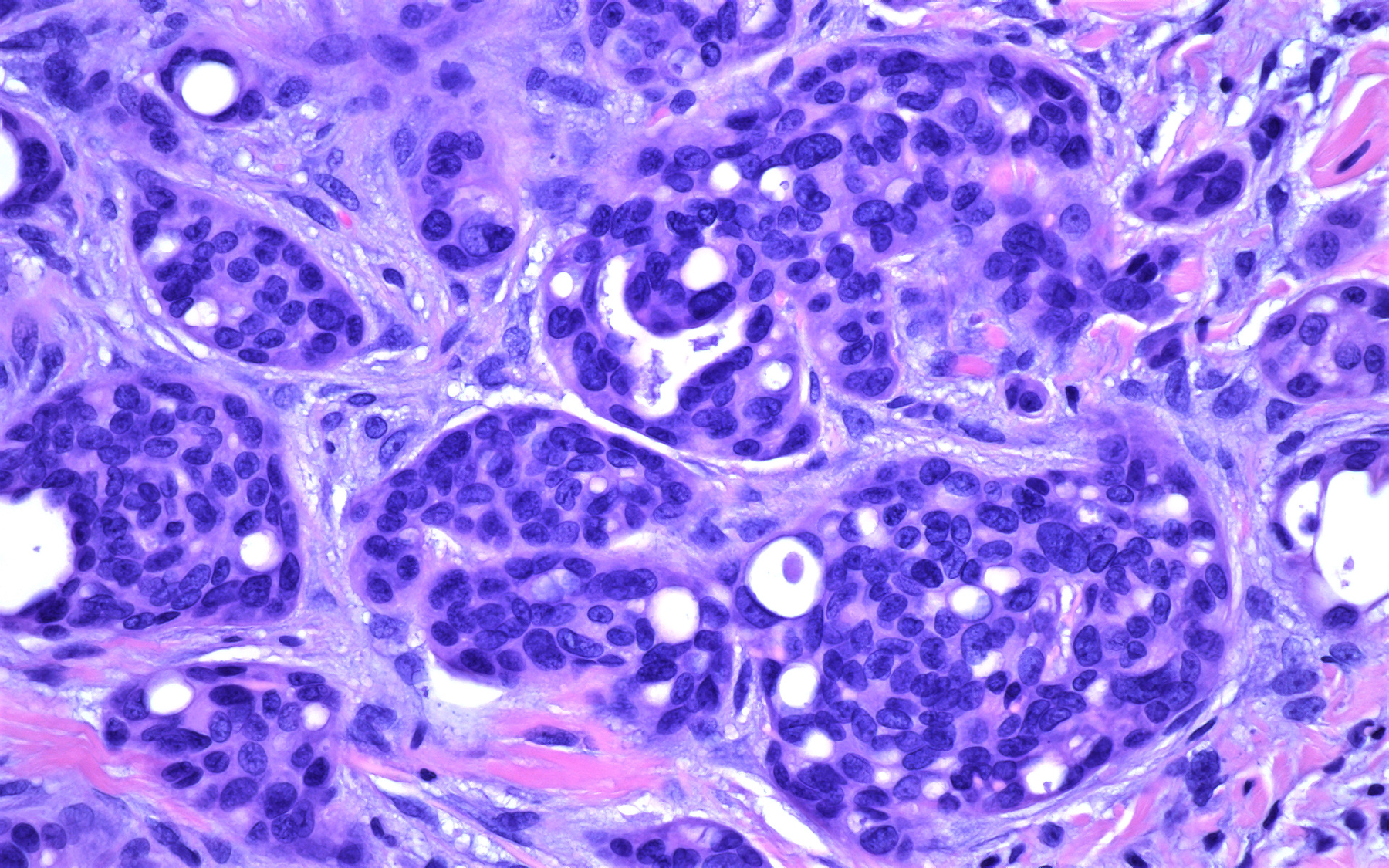
(**a**)**. Hematoxylin and eosin staining for primary cutaneous cribriform apocrine carcinoma at 20*×* magnification.** A nodular, well-circumscribed, nonencapsulated, pandermal tumor with a border abutting the underlying subcutaneous tissue, without connection to the overlying epidermis or adnexal structures, and a desmoplastic stroma is demonstrated. Lymphoid aggregates are appreciated in the periphery. (**b**) **Hematoxylin and eosin staining for primary cutaneous cribriform apocrine carcinoma at 40*×* magnification.** Characteristic anastomosing solid and cribriform nests, tubules, and cords of mildly atypical epithelial cells with eosinophilic cytoplasm and formation of glandular lumina. (**c**) **Hematoxylin and eosin staining for primary cutaneous cribriform apocrine carcinoma at 200*×* magnification.** Intratumoral lumina were characterized by size variations, attenuation of the surrounding epithelium, narrow anastomosing intraluminal bridges, and occasional micropapillary projections. (**d**) **Hematoxylin and eosin staining for primary cutaneous cribriform apocrine carcinoma at 400*×* magnification**. Mildly atypical cells with round or oval, hyperchromatic, basophilic, pleomorphic nuclei.

**Figure 2.**
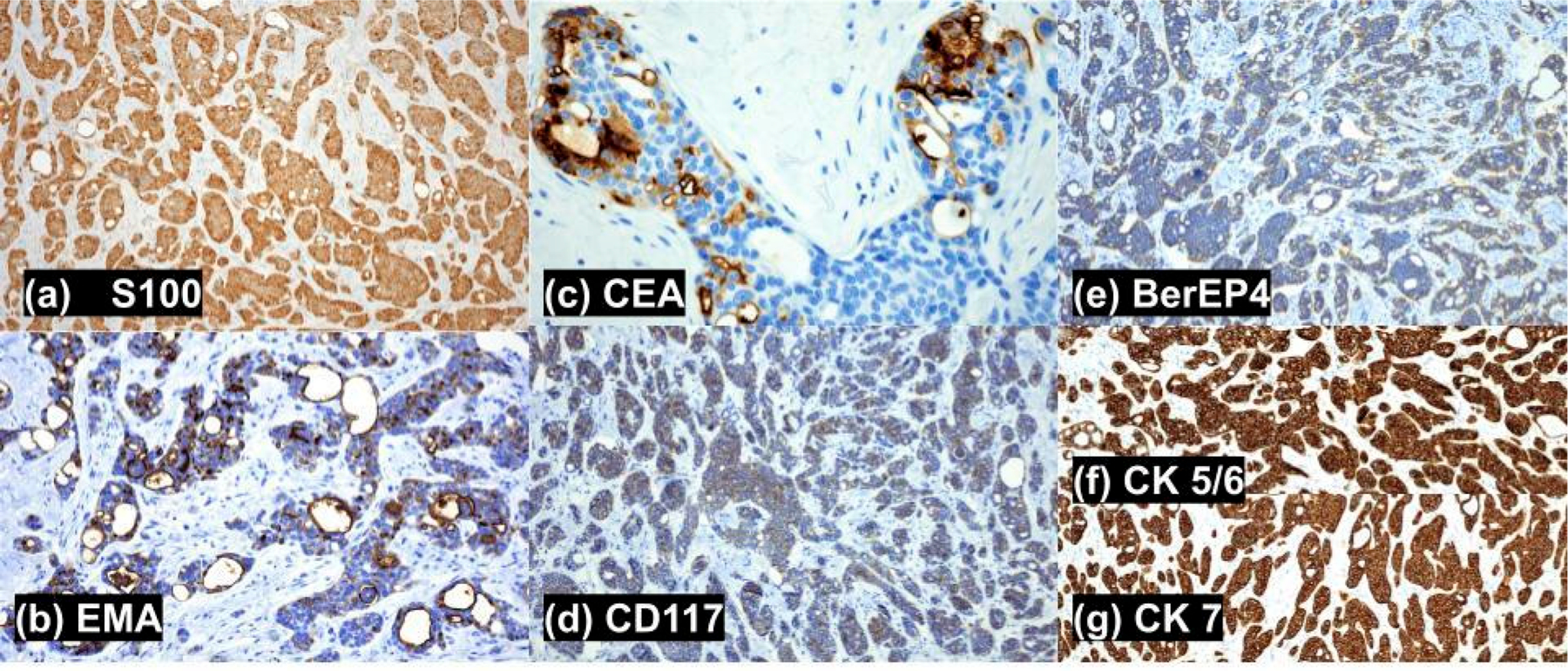
Positive Histopathologic Staining Patterns for primary cutaneous cribriform apocrine carcinoma (magnification). The specimen stained positive for the following stains: (**a**) S100 (100*×*), (**b**) EMA (200*×*), (**c**) CEA (400*×*), (**d**) CD117 (100*×*), (**e**) BerEP4 (100*×*), (**f**,**g**) CK 5/6/7 (100*×*).

A punch biopsy was obtained. Histopathological assessment revealed a nodular, well-circumscribed, nonencapsulated, pandermal tumor with a border abutting the underlying subcutaneous tissue, without connection to the overlying epidermis or adnexal structures, and a desmoplastic stroma. Characteristic anastomosing solid and cribriform nests, tubules, and cords of mildly atypical epithelial cells with eosinophilic cytoplasm and formation of glandular lumina (Figure 1a–d) were appreciated. Intratumoral lumina were characterized by size variations, attenuation of the surrounding epithelium, narrow anastomosing intraluminal bridges, and occasional micropapillary projections (Figure 1c,d). Foci of apocrine secretion by the luminal epithelium were appreciated but rare. Necrosis and mitotic figures were scarce (4 per 10 hpf). Occasional single-cell necrosis was present, but tumoral necrosis was absent. No lymphovascular or perineural invasion was observed. Lymphoid aggregates were present at the periphery of the tumor. Immunohistochemistry stains were positive for CK1/3, CK5/6, CK7, D2–40, BerEP4, mammaglobin, AE1/AE3, CAM5.2, and CD117 (Figure 2). EMA, CEA, and S100 staining were positive (Figure 2). In totality, the histopathological features and immunohistochemical stains were consistent with a diagnosis of primary cutaneous cribriform carcinoma.

The papule was excised via wide local excision with 4 mm margins, and no evidence of recurrence was observed at the 1-year follow-up. The patient was successively lost to follow-up.

## 4. Search Methods

A literature search restricted to accessible articles of the English language was conducted using PubMed to identify articles related to primary cutaneous cribriform apocrine carcinoma, invasive ductal metastatic breast carcinoma, primary cutaneous adenoid cystic carcinoma, primary cutaneous secretory carcinoma. Search terms included the aforementioned diagnoses as individual entities. The articles were assessed for relevance. The main inclusion criteria were articles including histology staining patterns, utilized to compose Table 1. Additionally, articles that contributed novel information on clinical presentation and histopathological description for these diagnoses were included. Exclusion criteria were articles that did not contribute information on clinical presentation and histology characteristics previously found in other sources. Literature appraisal and extraction of information were performed by two independent reviewers. Full-text screening was conducted on all encountered articles. The final count of articles used was 38.

## 5. Discussion

PCCAC typically presents as a slow-growing solitary nodule, papule, or cyst on the extremity, but may also occur on the head, neck, or trunk [1–4]. This patient was one of the few cases in the literature noted to exhibit a PCCAC occurring on the trunk; there were previously 4 other cases reported to be localized to the trunk in the literature [2]. Neither location nor clinical morphology has been appreciated to correlate with differences in histologic presentation, IHC staining, or clinical outcome [2]. This patient also endorsed a pruritic papule, in contrast to the usually asymptomatic or occasionally tender clinical presentations in the literature [2]. There are no specific clinical features on physical examination that reliably raise suspicion for PCCAC, which may contribute to delays in diagnosis. Histopathological features include large, nodular, well-circumscribed, unencapsulated, pandermal nests of epithelial cells separated by a fibrous stroma that may abut or extend to the subcutis. As seen in our specimen, there is typically no connection to the overlying epidermis or adnexal structures (Figure 1a). Lesional cells demonstrate mild pleomorphism and large, round-to-ovoid nuclei with hyperchromasia, nuclear grooves, and eosinophilic cytoplasm. The standard of treatment in the literature is with wide local excision, though excisions utilizing Mohs micrographic surgery have recently been described [2]. Prognosis is significantly favorable as no signs of recurrence have been documented in the literature, ranging from 3 months to 18 years [2].

Although PCCAC is believed to be derived from an apocrine line of differentiation, definitive apocrine or eccrine differentiation has not been defined. In contrast to other apocrine carcinomas that are usually localized to the anogenital or axillary regions (flexural areas with a high density of apocrine glands), PCCAC usually occurs on the extremities (areas with scarce apocrine glands) [2]. Additionally, positive immunohistochemical staining for GCDFP-15 may indicate apocrine differentiation; PCCAC is usually negative for this stain [2]. Nevertheless, there are many characteristics that favor apocrine differentiation. The presence of small papillary projections from neoplastic basophilic cells occasionally present within tubules, as well as ductal staining patterns highlighting decapitation secretion and luminal secretion of periodic acid-Schiff positive substances, indicates apocrine differentiation. However, Arps et al. noted a lack of decapitation secretion in 4 out of 6 reported cases of PCCAC (although CEA and EMA staining was positive) [1]. Immunohistochemistry (IHC) stains are positive for CEA and EMA, indicating ductal structures and aiding in distinguishing from other items on the differential (metastatic carcinoma, secretory carcinoma, and adenoid cystic carcinoma) [1–3]. The nests of epithelial cells yield positive cytokeratin (CK 5/6/7 and AE1/AE3) staining. As mildly atypical and pleomorphic cells are appreciated in PCCAC, p63 may be positive in this diagnosis, indicating the presence of atypical cells within aggregates [2]. IHC also reveals the absence of a myoepithelial layer as seen in our specimen, via negative analysis for SMA, calponin, and sometimes, p63 [4]. The lack of a myoepithelial layer helps to distinguish PCCAC from other mimickers, such as adenoid cystic carcinoma and tubular apocrine adenoma, which will consistently demonstrate myoepithelial staining [1]. However, the presence of myofibroblasts within PCCAC specimens may occasionally result in positive SMA staining [2]. Other variable stains include ER (higher positivity rates than PR), PR, and S100. Our specimen expressed S100 positivity (which serves as an indicator of myoepithelial cells in breast, salivary glands, and sweat glands); however, S100 has also been reported to be negative in some cases [1–3,5,6]. Negative stains include GATA3, adipophilin, HER2/neu (w/occasional membrane positivity), CK20, and GCDFP-15, all of which may indicate the alternative diagnosis of a mammary, prostate, or salivary gland carcinoma. Necrosis and mitotic figures are absent or scarce (1–3 mitoses per 10 fields at *×*400) [1,2]. Histopathologic differentials include metastatic breast carcinoma, primary cutaneous adenoid cystic carcinoma, primary cutaneous secretory carcinoma, and tubular apocrine adenoma. It is important to differentiate PCCAC from metastatic adenocarcinomas (e.g., breast, salivary gland, or visceral adenocarcinoma) and adnexal tumors with overlapping histology by characteristic features on histology and IHC staining patterns (Table 1).

Clinical and radiologic information proves most useful when trying to distinguish PCCAC from metastatic breast carcinoma, invasive ductal type (MMDAC). The histopathologic findings of primary cutaneous cribriform apocrine carcinoma (PCCAC) are often indistinguishable from metastatic breast carcinoma, invasive ductal type. Both tumors display poorly circumscribed dermal nests of infiltrative glandular, tubular, papillary, and tubulopapillary structures that may invade into the subcutis. The cellular morphology and level of mitoses and necrosis are similar in both diagnoses. In contrast to PCCAC, a cutaneous metastatic breast carcinoma may stain positive for GATA3 (which marks luminal cell differentiation in the breast and bladder), adipophilin (a marker for poor prognosis in breast malignancy), HER2/neu, and GCDFP-15 [6–8]. Unlike MMDAC, PCCAC will usually stain positive for CK 5/6, mammaglobin (at higher rates than MMDAC), D2–40, CD117, CEA, and occasionally p63 [6,8]. Although both tumors can stain positive for S100 and ER, PCCAC holds a greater tendency to exhibit positive staining throughout the literature [7,8]. While both tumors are usually negative, metastatic breast carcinoma, invasive ductal type, can sometimes be distinguished by PR and CK20 positivity (which indicates breast malignancy) [6,7].

Primary Cutaneous Adenoid Cystic Carcinoma (PCACC) tends to develop as a painful, slow-growing nodule or plaque with an ulcerated, cystic, or indurated appearance. It is usually located on the scalp (head and neck region), in a female in the sixth decade. In contrast to the well-circumscribed architecture of PCCAC, histopathology reveals a deep dermal tumor, often with subcutaneous extension of poorly circumscribed lobules, islands, or cords of basaloid epithelioid cells. Epidermal involvement is unusual. The nests of basaloid cells with cribriform and tubular patterns will feature abundant mucin in cysts and between cells, with more uniform nuclei surrounding the pseudo lumina. The lack of basaloid cells disposed in peripheral palisades in an adenoid-cystic pattern without connection to the overlying epidermis helps to distinguish primary cutaneous cribriform apocrine carcinoma from PCACC [7,8]. Like PCCAC, PCACC also has rare mitosis, though frequent perineural invasion and small true ducts with myoepithelial cell differentiation are characteristic of PCACC [1]. Both tumors stain positive for D2–40, CK 5/6, CK7, BerEP4, EMA, CEA, AE1/AE3, CAM 5.2, mammaglobin, CD117, can show ER and PR positivity, and are negative for GCDFP-15 and HER2/neu. Unlike PCCAC, PCACC will often stain positive for S100, p63, SMA, and calciponin (myoepithelial differentiation), as well as GATA3, adipophilin, and show diffuse positivity for CD117.

Primary Cutaneous Secretory Carcinoma (PCSC) primarily affects females in the fifth decade of life as a solitary, intradermal nodule in the axilla. PCSC will show a well-circumscribed, unencapsulated intradermal arrangement of neoplastic cells into solid, papillary, tubular, or microcystic patterns, containing conspicuous intraluminal secretions. Notably, PCSC has a sclerotic stroma. Back-to-back proliferation and cuboidal neoplastic cells characteristically distinguish it from PCCAC [1]. The cuboidal neoplastic cells are characterized as bubbly PAS-positive, diastase-resistant, eosinophilic secretions, with vacuolated cytoplasm, and bland oval to round nuclei. In contrast, PCCAC presents with cribriform areas with empty spaces or very discrete signs of apocrine secretion and lacks the typical cystic spaces with colloid-like secretion seen in PCSC [7,9]. Nuclear pleomorphism is milder in comparison to PCCAC, though both carcinomas lack neural invasion and abundant mitoses. Like PCCAC, PCSC also stains positive for S100 (more often than PCCAC), EMA, mammaglobin, BerEP4, AE1/AE3, CK 5/6, CK7, CD117, D2–40, and is negative for SMA. Both tumors offer variable positive staining for p63 and calciponin. PCSC has variable staining for CAM5.2 and GCDFP-15, while PCCAC will stain positive and negative, respectively. Unlike PCCAC, PCSC will stain positive for GATA3 and adipophilin, and hold negative staining results for CEA, ER, PR, and HER2/neu. Finally, the ETV6-NTRK3 gene fusion is frequently reported in the literature as a principal identifier for secretory carcinomas, staining positive approximately 80% of the time. PCSC is distinguished by its positive staining of the NTRK3 fusion gene.

Tubular Apocrine Adenoma (TA) usually presents in the scalp, with other areas of occurrence including the face, axilla, and genitalia [2,7]. The typical anatomical locations are mostly hair-bearing areas, where normal apocrine glands are abundant [2,7]. The clinical exam demonstrates an asymptomatic, lobulated, slow-growing nodule, which is fixed to the dermis [2,7]. Histopathology reveals a lobular pattern of tubular structures located in the dermis, sometimes extending to the subcutis [2,7]. The tubules contain typical apocrine epithelial cells, some with hyaline or clear cell differentiation [5,7]. The tubules are composed of an inner tall columnar cell layer and an outer cuboidal cell layer, which show decapitation secretion, with eosinophilic cytoplasm and round nuclei [2,7]. Sometimes, the inner columnar layer features comedo-like channels that extend into the epidermis [2,7]. TA can show well-circumscribed, dilated cystic spaces with micro-papillae, focal intraluminal bridging, and absent perineural invasion, mimicking PCCAC. However, this condition lacks cytologic atypia and mitotic activity completely. TA can be distinguished from PCCAC by the presence of a myoepithelial layer around the tumor nests and uniform nuclei. The tubular dominance, paucicellular fibrous stroma, attenuated epithelium, and conspicuous amounts of intraluminal decapitation secretions distinguish TA from PCCAC. Like PCCAC, TA stains positive for CK 5/6, CK7, AE1/AE3, mammaglobin, EMA and CEA, BerEP4, D2–40, CAM5.2, and CD117, has variable staining for S100, and can have positive stains for p63, calciponin, and ER. Unlike PCCAC, TA can be distinguished by positive staining for SMA, GATA3, GCDFP-15, and S100. Negative stains TA shares with PCCAC are inclusive of adipophilin, HER2/neu, and BRST2. Unlike PCCAC, TA stains negative for PR.

## 6. Conclusions

It is important to accurately distinguish the histological attributes of PCCAC from its corresponding histological differential, especially metastatic malignancy. This case and literature review contribute to a growing body of literature, serving to effectively differentiate the histological features and staining profile of the differential diagnosis for PCCAC. Future studies should aim to definitively establish apocrine differentiation of PCCAC, as well as outline distinct mechanisms relating this nature to observed IHC staining patterns.

## 7. Limitations

The patient was lost to follow-up after the 1-year evaluation, precluding assessment of PCCAC outcomes beyond this interval. Regarding presentation of evidence, the authors were not able to include images of all reported positive stains (CK1/3, D2–40, mammaglobin, AE1/AE3, and CAM5.2).

## Figures and Tables

**Table 1.** Histopathologic Differential Diagnosis Staining Patterns for Primary Cutaneous Apocrine Cribriform Carcinoma (pCCAC) [1, 2, 3, 4, 5, 6, 7, 8, 9, 10, 11, 14, 15, 16, 17, 19, 20, 21, 24, 26, 27, 28, 29, 30, 31, 32, 33, 34, 35, 36, 37, 38]

Stain	Cutaneous Apocrine Cribriform Carcinoma (pCCAC)	Metastatic breast carcinoma, invasive ductal type (MMDAC)	Cutaneous Secretory Carcinoma (pCSC)	Cutaneous Adenoid Cystic Carcinoma (pCACC)	Tubular Apocrine Adenoma (TA)
GATA 3	negative	positive	positive	positive	positive
Adipophilin	negative	Mostly positive	positive	positive	Negative
HER2/neu	negative	Mostly positive	negative	variable	negative
CD117	Mostly positive	negative	positive	positive	positive
p63	variable	negative	variable	Positive	positive
D240	positive	negative	positive	positive	positive
SMA	negative	negative	Mostly negative	Mostly positive	positive
CK 7	Mostly positive	Mostly negative	positive	Mostly positive	positive
CK 5/6	positive	Mostly negative	positive	positive	positive
S 100	variable	variable	positive	Mostly positive	Mostly positive
EMA	positive	N/A	Mostly positive	positive	positive
GCDFP-15	negative	Mostly positive	Mostly positive	negative	Mostly positive
CEA	positive	Mostly negative	Negative	Mostly positive	positive
ER+	variable	variable	negative	variable	positive
PR+	variable	variable	negative	Mostly negative	negative
AE1/AE3	Mostly positive	N/A	positive	positive	positive
CAM5.2	Mostly positive	N/A	Mostly positive	positive	positive
calciponin	Variable	N/A	Mostly positive	positive	positive
mammaglobin	positive	Mostly positive	positive	positive	positive

## Data Availability

The original contributions presented in this study are included in the article. Further inquiries can be directed to the corresponding author.
